# 2023 ISCB innovator award: Dana Pe’er

**DOI:** 10.1093/bioinformatics/btad318

**Published:** 2023-06-30

**Authors:** Christiana N Fogg, Diane E Kovats, Martin Vingron

**Affiliations:** Freelance Writer, Kensington, MD, United States; International Society for Computational Biology, 525K East Market Street, RM 330, Leesburg, VA 20176, United States; International Society for Computational Biology, 525K East Market Street, RM 330, Leesburg, VA 20176, United States; Max-Planck-Institute for Molecular Genetics Computational Molecular Biology, Ihnestr. 73, Berlin 14195, Germany

The annual ISCB Innovator Award recognizes a scientist who is within two decades of completing her or his graduate degree and has made profound contributions to the field of computational biology or bioinformatics. The 2023 ISCB Innovator Award winner is Dr. Dana Pe’er, Chair and Professor of Computational and Systems Biology at the Sloan Kettering Institute and Howard Hughes Medical Institute Investigator. She will receive her award and deliver a keynote address at the 2023 Joint ISMB/ECCB meeting in Lyon, France this July.


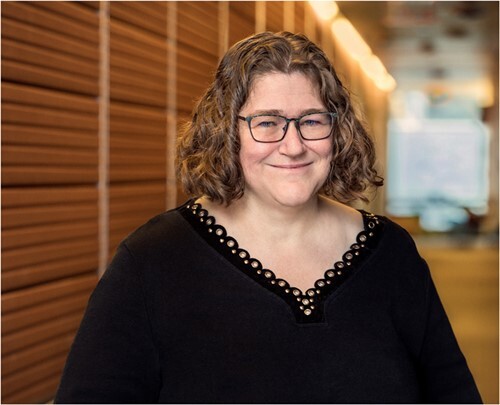
 *Dana Pe’er, Sloan Kettering Institute.*

## Dana Pe’er: from mass cytometry to plasticity

Dana Pe’er has cultivated her love of mathematics since her childhood in Israel, recalling lessons from her father that revealed the beauty of mathematical logic (Fogg and Kovats 2014). As a high school student, she had her first hands-on experience in the lab of Idan Segev at the Hebrew University of Jerusalam, where she used mathematical modeling to understand subthreshold oscillations in neurons. This project exposed Pe’er to mathematical applications for biological questions, planting seeds for her future research interests. Although Pe’er had contemplated a degree in neurobiology, her curiosity in genomics and bioinformatics was kindled after listening to a mesmerizing talk by Eric Lander describing the onset of the human genome project. She completed her bachelor’s degree in mathematics and master’s and PhD degrees in computer science at Hebrew University. Pe’er’s PhD research mentor Nir Friedman revealed to her the power of statistical machine learning in interpreting complex biological data. Pe’er also came to appreciate the importance of having a strong foundation in abstract biological concepts through her collaboration with fellow graduate student Aviv Regev. After graduate school, Pe’er pursued her postdoctoral studies in the lab of George Church at Harvard University, where she learned to wrestle with the ambiguities of wet lab biology. Her perspective also shifted from asking, “What type of computation can I do for this data?” and learned to ask instead, “What data do I need to answer a biological question I am passionate about?” (Fogg and Kovats 2014) Pe’er also gained invaluable informal mentorship during her PhD and postdoc from Daphne Koller, who not only instructed her in the importance of good modeling assumptions but also provided critical career advice as she prepared to become an independent researcher. Pe’er launched her own lab in 2006 at Columbia University in the Department of Biological Sciences and Systems Biology.

During her postdoc, Pe’er realized the power of single cells and that inter-cell variability can be exploited for regulatory circuit reconstruction. Single cell approaches accelerated as she launched her own lab at Columbia University in part through pioneering research with Garry Nolan, for which she developed critical aspects of the computational foundation for single-cell data analysis. These studies opened the floodgates of data science to immunologists and Pe’er was uniquely positioned to take on these studies at the juncture of computational biology and immunology. Pe’er introduced the single-cell field to large-scale analysis with the conceptual framework in which cell phenotypes are constrained to geometric manifolds corresponding to landscapes of possible cell states. This established a now-dominant paradigm that models cell state transitions in development and disease as continuous processes rather than discrete toggles. By leveraging asynchrony in cell states, she demonstrated that it is possible to infer continuous pseudotime trajectories, which provide dynamics from a single sample and generated fundamental knowledge in numerous development, immunology, cancer, and regenerative medicine studies. Pe’er also developed the neighbor-graph-based representation of the phenotypic manifold that serves as the field standard, and guided her development of widely used methods for identifying cell types, visualizing the manifold, deriving pseudotime trajectories, identifying lineage bifurcations, and quantifying developmental potential.

As Pe’er became an established PI, she was more involved in the greater computational biology community, including serving as a founding member of the Human Cell Atlas (HCA) Project. She played pivotal roles in formulating the vision of the HCA and has been a key driver of the computational direction of the HCA, especially through her role as co-chair of the Analysis Working Group within the HCA. In 2016, Pe’er moved her lab to the Sloan Kettering Institute (SKI), where she became Chair of the Computational and Systems Biology Program and Scientific Director of the Alan and Sandra Gerry Metastasis and Tumor Ecosystems Center. In this new home, she said, “My focus on biology completely changed. I was in a new environment with great peers, like Sasha Rudensky and Scott Lowe, who also became my teachers.” Pe’er was also given the responsibility of developing the Single Cell Research Initiative at SKI, which has flourished by harnessing the power of single cell analysis to address fundamental cancer and immune system questions. Pe’er’s own team has published seminal findings in cancer research that revealed the complexity of the tumor immune microenvironment and has nurtured her fascination with cell plasticity. She said, “I want to understand how cells work in tissues. Plasticity helps cells respond to their neighbors, and during development, cells lean on their plasticity to form tissues.” Some of Pe’er’s most recent work has shown how the plasticity of tumor cells allow them to hijack and mimic programs of embryonic organogenesis, which ultimately drives metastasis. Although Pe’er gets excited about tackling biology questions, she still loves being in the trenches of algorithm development and troubleshooting technical problems. She considers questions related to plasticity to be particularly well-suited to computational approaches and said gleefully, “These questions require so much math, and math is my playground.”

In 2022, Pe’er was awarded an appointment as an HHMI investigator in recognition of fundamental studies on cellular plasticity and how it shapes many biological processes. This recognition validates the broader impacts of single cell studies and solidifies Pe’er’s role as a leader of the field. Pe’er’s impressive publication record and numerous awards further highlight the many contributions she has made to computational biology, including her recognition with the 2014 ISCB Overton Prize. Pe’er feels deeply honored to be recognized with the 2023 ISCB Innovator Award, particularly because it comes from her computational biology peers. Pe’er’s infectious enthusiasm for current research projects is certain to lead to many new algorithms and insights in the future.
